# Natural-Series Radionuclides in Traditional Aboriginal Foods in Tropical Northern Australia: A Review

**DOI:** 10.1100/tsw.2004.6

**Published:** 2004-02-26

**Authors:** Paul Martin, Bruce Ryan

**Affiliations:** Environmental Research Institute of the Supervising Scientist, Darwin, NT, Australia

**Keywords:** natural-series radionuclides, bioaccumulation, Australian Aboriginal, tropics, uranium mining

## Abstract

This paper gives a review of available information on natural-series radionuclides in traditional Aboriginal foods of northern Australia. Research on this topic has been carried out primarily for radiological impact assessment purposes in relation to uranium mining activities in the region. Many of the studies have concentrated on providing purely concentration data or concentration ratios, although more detailed uptake studies have been undertaken for freshwater mussels, turtles, and water lilies. The most-studied radionuclides are U and Ra. However, dose estimates based on current data highlight the importance of Po, particularly for the natural (nonmining-related) dose. Data on uptake by terrestrial flora and fauna are scarce in comparison with aquatic organisms, and this knowledge gap will need to be addressed in relation to planning for uranium minesite rehabilitation.

## INTRODUCTION

This paper gives a review of available information on natural-series radionuclides in traditional Aboriginal foods of northern Australia. Research on this topic has been driven by the coincidence of a number of factors. These include: the presence of uranium mining activities over several decades, particularly within World Heritage listed Kakadu National Park and western Arnhem Land, areas which are occupied by Aboriginal people living a semi-traditional lifestyle; the monsoonal tropical climate of the region; early experience of environmental problems resulting from acid mine drainage at the Rum Jungle mine; and recommendations for research made by the Ranger Uranium Environmental Inquiry[1]. The primary focus of the research has been on providing information useful for radiological impact assessment purposes, particularly in relation to intake of uranium series radionuclides by Aboriginal people.

## STUDY AREA

Fig. 1 shows the general region containing uranium mining and milling operations in the "Top End" of the Northern Territory of Australia. In this context, the term "Top End" essentially refers to that northernmost part of the Northern Territory which experiences a tropical monsoonal climate.

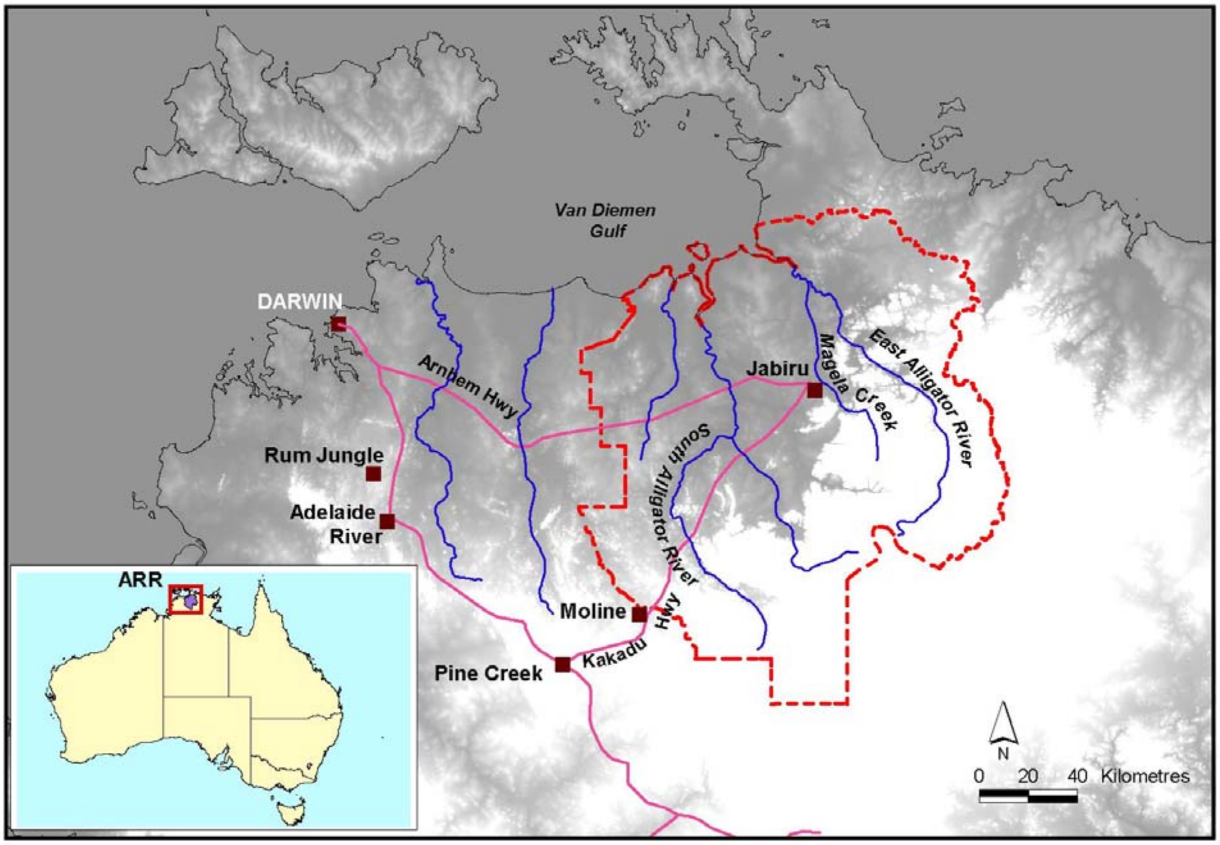

**FIGURE 1.** Maps of Australia and of the Top End, showing the locations of the Alligator Rivers Region, and the location of the Rum Jungle mine and Moline mill sites.

Apart from Rum Jungle and Moline, all of these uranium mines, mills, and known major orebodies lie within the Alligator Rivers Region (ARR, see Fig. 2). The ARR occupies an area of approximately 28,000 km$^2$ lying to the east of Darwin. It comprises and is roughly defined by the catchments of the West Alligator, South Alligator, and East Alligator Rivers. The eastern portion of the ARR is approximately 8,000 km$^2$ and is contained within the Arnhem Land Aboriginal reserve. The remainder encompasses Kakadu National Park and the three mineral leases of Ranger, Jabiluka, and Koongarra that lie within, but are not a part of, the national park.

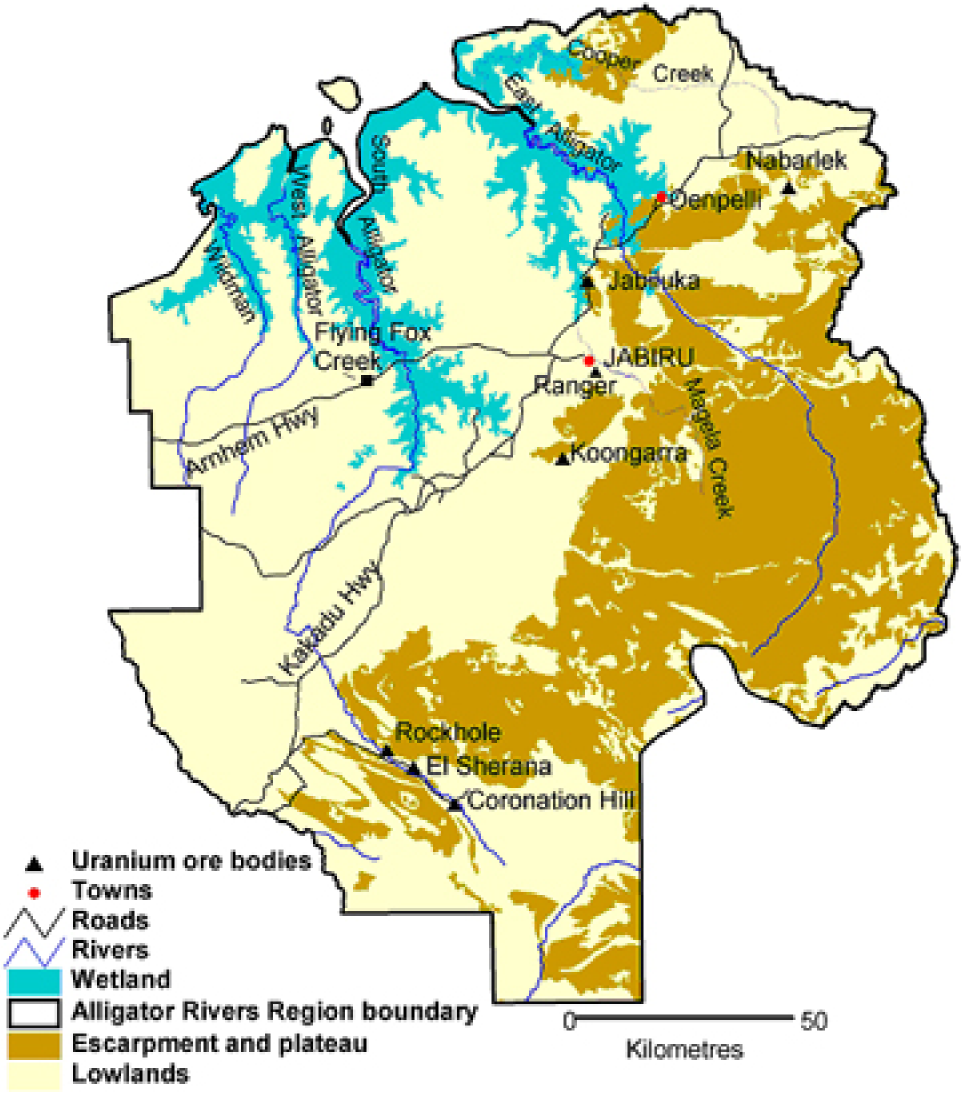

FIGURE 2. **The Alligator Rivers Region.**

## History of Uranium Mining in the "Top End"

The Rum Jungle minesite is 64 km south of Darwin. Mining of copper, lead, and silver began in the area in the early 20th century, but large-scale mining operations did not commence until after the discovery of uranium in the bed of the East Finniss River in 1949 by a local prospector[2].

The treatment plant produced approximately 3,500 tonnes of uranium and 20,000 tonnes of copper until cessation in 1971. Mining caused severe impacts on the environment of the minesite and the East Branch of the Finniss River. This arose primarily as a consequence of acid rock drainage processes developing in the waste rock dumps, leading to the mobilisation of metals (including uranium, copper, nickel, zinc, and manganese). The Australian Government undertook remediation of the site during the 1980s, and monitoring of the site is continuing[3].

Several prospects were mined in the South Alligator Valley in and around what is now known as Stage 3 of Kakadu National Park. The first of these prospects was discovered in 1953[4]. There were two treatment plants in the area drawing upon ore from different mines[5].

In 1958, United Uranium purchased the North Hercules gold plant at Moline, 50 km east of Pine Creek (about 65 km from the South Alligator mines). The plant was commissioned with an annual capacity of about 130 tonnes $U_3O_8$ in May 1959[5].

In 1970, a uranium orebody was discovered at Nabarlek 15 km east of Oenpelli (Gunbalanya), near Cooper Creek (a tributary of the East Alligator River). The Nabarlek 1 orebody was mined out in just over 4 months during the dry season of 1979. With an average ore grade of 2%, 600,000 tonnes of ore were stockpiled with processing beginning in 1980 and continuing for the next 9–10 years[5].

Ranger is the only operating uranium mine in the Top End at present. The Ranger orebodies #1 and #3 are about 1,200 m apart, and are situated near Magela Creek, a tributary of the East Alligator River. Mining of Ranger #1 occurred between 1980 and 1994 using an open-cut method. Complete mining of approximately 20 million tonnes of ore from this orebody at an average grade of 0.327% required the removal of about 60 million tonnes of waste rock and very low-grade mineralised material. Open-cut mining of Ranger #3 commenced in 1997 and milling of this ore is expected to continue to 2014. Fig. 3 shows the region around the Ranger minesite, where much of the sampling for studies discussed in this paper has occurred.

The Koongarra prospect lies some 30 km south of Ranger. The upper orebody has proved and probable ore reserves containing 14,500 tonnes of uranium oxide with an average grade of almost 0.8% $U_3O_8$. This ore would be accessible by open-pit mining and there is associated gold within the orebody[5].

The Jabiluka 1 uranium deposit was discovered in 1971. In 1973, further drilling located the larger Jabiluka 2 uranium orebody about 1 km to the east. Jabiluka lies on the edge of the Magela Creek floodplain, and is 20 km to the north of Ranger. Jabiluka 2 has reserves in excess of 160,000 tonnes of uranium oxide, and is one of the world's larger high-grade uranium deposits[5].

## Climate

The climate is tropical monsoonal and characterized by intense rainfall during the wet season (November to April), followed by an almost rainless dry season (May to October). At Jabiru airport, the mean annual rainfall and annual pan evaporation recorded between 1971 and 1998 were 1,480 and 2,615 mm, respectively[6]. Almost 96% of rainfall occurs in between November and April and almost 60% in the 3 months of January, February, and March.

The Inter Tropical Convergence Zone extends into Northern Australia in the summer months, characterized by the inland of Australia being intensively heated resulting in a flow of tropical maritime air over the region[7] producing strong convective rainfall. These convective storms result in high-intensity rainfalls for short periods of time during the wet season. The wet season monsoonal winds originate in equatorial regions and move in from the northwest across the coast bringing hot, humid, unstable conditions. Thunderstorms can be common along the coast with an average of 60–80 thunder days each year, which become less frequent further south[8]. Monsoonal depressions cause heavy rains and tropical cyclone activity.

## Traditional Aboriginal Foods and Estimation of Radiological Impact

Aboriginal people of northern Australia obtain food by a combination of commercial purchases (primarily of items imported from outside the region) and customary harvesting of both native and introduced flora and fauna ("bush foods"). In an anthropological study carried out in 1979–80 at Momega in western

Arnhem Land, Altman[9] found that 46% of total kilocalories and 81% of total proteins came from bush foods. He identified over 80 floral species used as bush foods, and in total 170 species of flora and fauna were observed being consumed. The majority of these are native species that are not used in agriculture, and so very little information on element or radionuclide concentrations in the edible portions is available. A sample of a single species will generally have several edible parts (e.g., various organs of an animal). For some species, Aboriginal people may reserve different parts of the animal or plant for different people. The reasons for this may include land ownership, social standing in the clan, age group, gender, and personal totem responsibilities.

Taking into consideration the number of radionuclides that could be important from a dose assessment perspective (these include isotopes of uranium, thorium, radium, lead, polonium, and actinium), it can be seen that the full matrix of bioaccumulation pathways that are of interest is very large. In the case of work undertaken in the ARR over the last 2 decades, researchers have generally addressed this problem by taking one or more of the following approaches:

- Initially targeting aquatic organisms, as these were expected to be the most important during the operational phase of uranium mining.
- Undertaking detailed radionuclide uptake studies on radionuclides and species for which there is evidence that they are important from a dose perspective (in particular uptake of $^{226}$Ra by freshwater mussels, turtles, and water lilies).
- Using the concentration ratio approach to assess other radionuclide/bush food combinations.
- Carrying out long-term monitoring of specific radionuclide/food combinations. Such monitoring has been primarily carried out by the mining companies[10].

The concentration ratio (CR) or concentration factor (CF) method of dose assessment has been discussed in detail elsewhere[11,12,13,14,15]. The CR for a radionuclide in an organism is defined as the activity of the nuclide per unit weight of the organism, divided by the activity of the same nuclide per unit weight of substrate, where the substrate is the physical medium (e.g., water, food, or soil) from which the organism obtains the nuclide. Deficiencies in the CR approach are known to exist but, in general, its use is likely to be conservative provided locally derived values are used.

Comparison of concentration and CR data between different studies is not always possible because there is no standard either for the substrate or for the form of the analysed tissue or substrate (i.e., fresh weight or "wet weight" vs. dry weight of tissue; total vs. filtered water sample; wet vs. dry sediment or soil sample). It is important that researchers explicitly state this information in their reports in order that the data can be properly assessed and utilised.

## AQUATIC FLORA AND FAUNA

### Water Lily (*Nymphaea* species)

Water lilies from the genus *Nymphaea* are widespread and common in the Top End, typically growing in seasonally inundated areas with a water depth to about 2 m[16]. They are abundant in the floodplain backwater swamps and billabong fringes of the Magela Creek.

Accumulation of radionuclides by water lilies was recognised early as a potentially important pathway for radiological impact arising from releases of water from the Ranger mine[17]. Such accumulation can result from uptake from the water column (whether by absorption of dissolved radionuclides or deposition of fine suspended sediments or colloidal forms onto submergent surfaces) and uptake into roots and rhizome from the sediment in which the plant is rooted, with the possibility of subsequent translocation in the plant. Local Aboriginal people eat all parts of the plant including the rhizomes, flowers, seed heads, and certainly the stems, which are a favoured food[18].

Uptake of $^{226}$Ra by *Nymphaea* sp. was the subject of a series of studies by Twining[19,20,21,22]. He found that $^{226}$Ra concentrations in the root and rhizome are higher than in foliage, this being due primarily to surface accumulation. Little radium reached the pith of the rhizomes. Uptake of radium by the foliage was found to be primarily from the water rather than by translocation from the roots. For samples collected in the field, the distribution of radium and calcium concentrations in the foliage were strongly correlated. However, analysis of the ratio of radium to calcium in the plant, compared to extractable concentrations in the water and sediment, showed no correlation between foliage and supporting media, suggesting that different mechanisms were involved in accumulation of radium and calcium. In a laboratory experiment of uptake of $^{226}$Ra to leaves from an artificial Magela Creek water, a CR of 3,600 L $kg^{-1}$ dry weight (DW) was obtained[19]. Derived uptake and loss rate coefficients were 2.00 and 0.55 $day^{-1}$, respectively, although there was evidence of a second compartment for accumulation with a very low loss rate.

Pettersson et al.[23] measured concentrations of $^{234,238}$U, $^{228,230,232}$Th, $^{226}$Ra, $^{210}$Pb, and $^{210}$Po in root, rhizome, and foliage of *Nymphaea* sp. as well as sediment and water samples from four billabongs (permanent waterholes) on the Magela Creek system in order to obtain CRs for these radionuclides. Comparing concentrations in water lily parts and concentrations in sediment and water, they obtained better correlations with the sediment and concluded that this is the primary source of radionuclides to the plant. Despite the fact that the radionuclides studied represented five different elements, there was only a small variation in the derived CRs. For example, in the case of foliage, the CRs (waterlily fresh weight to sediment wet weight) for the eight radionuclides ranged between 0.0045 (for $^{232}$Th) to 0.014 (for $^{228}$Th). For roots and rhizomes the derived CRs were of the order of 0.01 to 0.03.

Hancock[24] extended the Pettersson et al.[23] study by carrying out similar measurements for six individual plants and associated sediment from one billabong. His results were broadly consistent with those of Pettersson et al.[23], although the derived CRs were about a factor of 5–7 lower. For foliage, the CRs relative to sediment followed the element order Ra > Po > Pb > U ≥ Th, with about an order of magnitude difference between the CRs for Ra and Th.

## Freshwater Mussels (*Velesunio angasi*)

A large number of studies have been conducted on the freshwater mussel, *Velesunio angasi*, in coastal streams of the Northern Territory, particularly populations from the Magela Creek system. Humphrey and Simpson[25] carried out a comprehensive study of the autecology of the species, including development of a method for age determination using annular dark rings in the shells, which has provided a base for many of the subsequent studies.

Early research showed that populations of *V. angasi* in the Magela Creek system had very high flesh concentrations of $^{226}$Ra[26]. The species is also a significant food source for some local Aboriginal groups. Consequently, the potential radiological impact arising from release of uranium mine and mill waters in the region is believed to be dominated by radium uptake by mussels[27,28], and this has been the major reason for the research undertaken on radionuclide uptake mechanisms by this species.

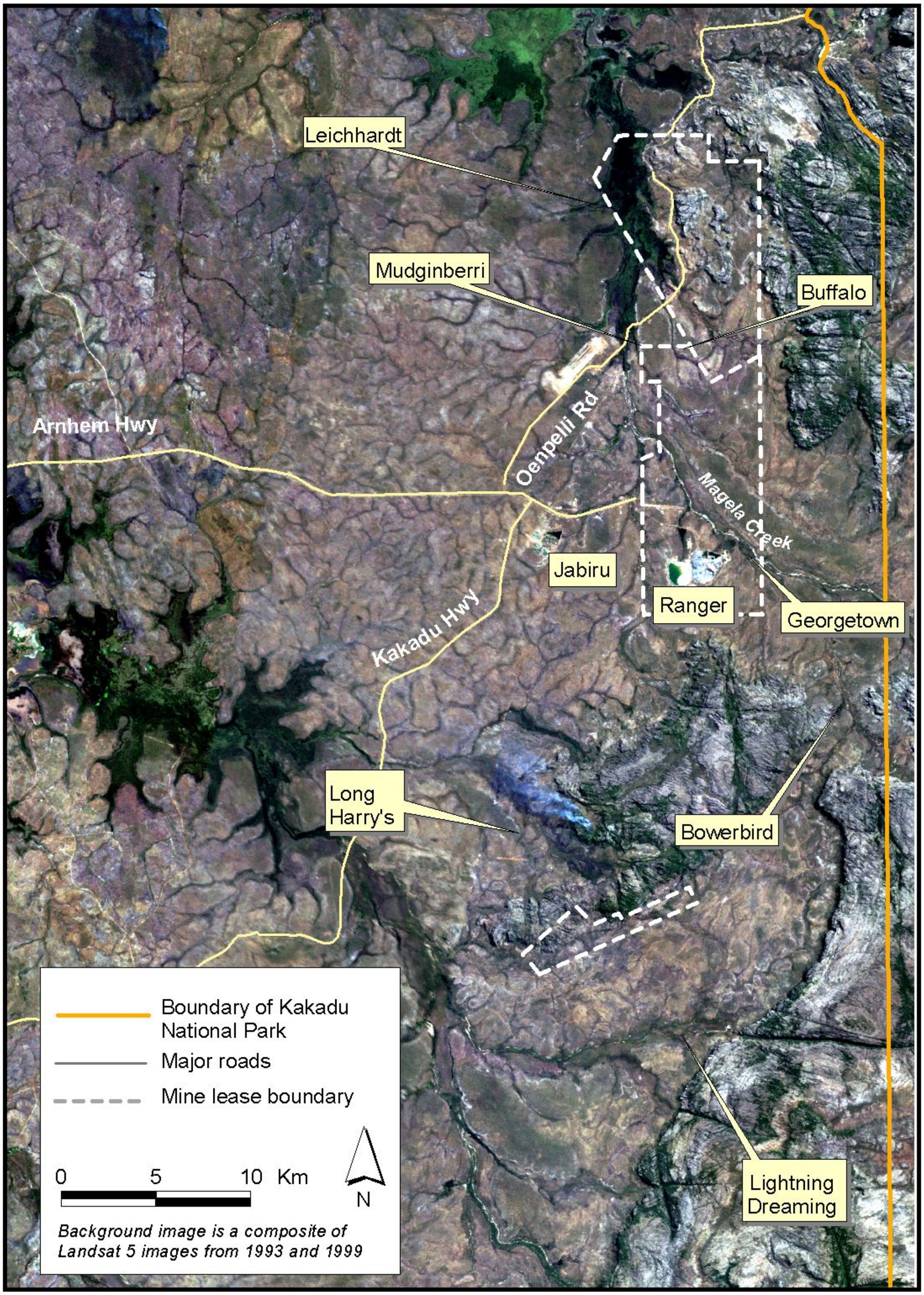

**FIGURE 3.** The region around the Ranger uranium minesite.

**TABLE 1**
**Concentrations of $^{226}$Ra and $^{228}$Ra/$^{226}$Ra Activity Ratios for *V. angasi* Flesh Samples Collected from Various Locations in the ARR, 1983, Together with Data for Ca and Mg Concentration in Billabong Water[30], and $^{226}$Ra Concentration and $^{228}$Ra/$^{226}$Ra Activity Ratios for Billabong Surface Sediment**

| Site | Location Relative to U Mine | Mussel* $^{226}$Ra (Bq kg$^{-1}$ DW) | Mussel* $^{228}$Ra/$^{226}$Ra | Water** Ca Range (*n*) (mg L$^{-1}$) | Water** Mg Range (mg L$^{-1}$) | Sediment*** $^{226}$Ra (Bq kg$^{-1}$ DW) mean (s.d.) | Sediment*** $^{228}$Ra/$^{226}$Ra mean (s.d.) | No. Sediment Samples |
|---|---|---|---|---|---|---|---|---|
| *Magela catchment* | | | | | | | | |
| Bowerbird billabong | 17 km upstream Ranger | 3,440 | 0.43 | 0–0.5 (15) | 0.2–0.9 | 8.7 (0.9) | 1.02 (0.41) | 2 |
| Georgetown billabong | 2 km downstream Ranger | 2,180 | 0.22 | 0.1– 0.9 (28) | 0.3–1.9 | 278 (138) | 0.290 (0.081) | 10 |
| Mudginberri billabong | 11 km downstream Ranger | 2,560 | 0.26 | 0.2–1.1 (31) | 0.3–3.5 | 169 (40) | 0.374 (0.086) | 6 |
| Buffalo billabong | 12 km downstream Ranger | 2,770 | 0.25 | 0.2–0.5 (9) | 0.4–0.7 | — | — | |
| Leichhardt billabong | 24 km downstream Ranger | 920 | 0.56 | 0.2–5.4 (32) | 0.4–26.5 | — | — | |
| *Cooper catchment* | | | | | | | | |
| Cooper Creek | 2 km upstream Nabarlek | 1,190 | 0.69 | — | — | — | — | |
| Cooper Creek | 12 km downstream Nabarlek | 380 | 0.60 | — | — | — | — | |
| Cooper Creek | 14 km downstream Nabarlek | 1,550 | 0.55 | 0.6–1.8 (7) | 0.9–3.0 | — | — | |
| *Nourlangie catchment* | | | | | | | | |
| Lightning dreaming | | 1,450 | 0.32 | — | — | — | — | |
| Long Harry's billabong | | 640 | 0.38 | — | — | — | — | |
| *West Alligator catchment* | | | | | | | | |
| Flying Fox sand pit | | 73 | 1.2 | — | — | 4.0 ± 0.2 | 1.36 ± 0.08 | 1 |

* Mussel data from [29]. Dry: fresh weight ratios for mussel flesh are generally in the range 0.08–0.15.

** Ca and Mg concentrations in billabong waters are ranges (no. samples) reported in [30] for the period 1978–1981; data for Cooper Creek were for Nimbuwah Billabong.

*** Sediment results are for surface sediment samples collected by ***eriss*** over 1983–87, and analysed by gamma-ray spectrometry.

Table 1 shows results for $^{226}$Ra concentrations and $^{228}$Ra/$^{226}$Ra ratios for samples collected from a number of locations in the ARR in 1983 (each sample comprised the combined flesh of a number of mussels[29]). These data show that the high $^{226}$Ra concentrations are regional in nature rather than being confined to areas downstream of uranium mining operations. The $^{228}$Ra/$^{226}$Ra ratios for mussels and sediments from the three billabongs immediately downstream of Ranger were lower than for mussels from other billabongs, however this is to be expected as the ratios for soils of the Ranger area are naturally low.

By contrast with the high $^{226}$Ra and $^{228}$Ra concentrations in mussel flesh, specific alpha activities measured for shells of mussels from Georgetown billabong were only of the order of 40–60 Bq kg$^{-1}$[31].

Radium is stored in calcium phosphate granules in the mussel flesh[32,33]. Radium is retained in these granules over long periods, resulting in a long effective biological half-life in the mussel tissue. As can be seen in Table 1, decay of accumulated $^{228}$Ra (radioactive half-life = 5.75 years) over the life of the mussel results in lower $^{228}$Ra/$^{226}$Ra ratios in mussel tissue than in the billabong sediment. Measurements and modelling of $^{228}$Ra/$^{226}$Ra ratios vs. mussel age yielded an estimate for the radium biological half-life of 8.8 ± 1.0 years in mussels from Mudginberri billabong[34].

$^{226}$Ra uptake rates are inversely proportional to both calcium and magnesium water concentrations[35]. Consequently, the high flesh radium concentrations in mussels from ARR creek systems are an outcome of the low calcium and magnesium concentrations in the water and the long biological half-life of radium in the mussel. This explains why the highest $^{226}$Ra concentrations in mussels shown in Table 1 were obtained for Bowerbird, a billabong with low $^{226}$Ra concentrations in the bottom sediments but also low calcium and magnesium concentrations in the water.

As a result of the long biological half-life, radium concentrations are positively correlated with mussel age, as well as with other age-correlated parameters such as shell length and tissue mass, and with concentrations of the alkaline earths calcium, magnesium, and barium[36,37,38,39]. Mussel size and sex have little or no effect on the rates of uptake of $^{226}$Ra per gram of tissue[35].

The biological half-life for $^{210}$Pb in *V. angasi* is long; one experimental study showed no significant reduction in tissue concentrations over 160 days[40]. As a consequence of this, plus the fact that there is some support from its $^{226}$Ra progenitor, $^{210}$Pb is accumulated in the tissue[34,38]. By contrast, the biological half-life for uranium is only of the order of a few days[41].

Concentrations of $^{210}$Po in the mussel flesh are significantly higher than those of its progenitor $^{210}$Pb[34], resulting in a high CR (see Table 2). Since the radioactive half-life of $^{210}$Po is only 138 days, the high concentrations imply that uptake rates are high for polonium.

**TABLE 2**
**CRs (L kg$^{-1}$) for Radionuclides of the Uranium Series in *V. angasi*, Determined from Samples Collected in the ARR**

| $^{226}$Ra | $^{210}$Pb | $^{210}$Po | $^{234,238}$U | $^{230}$Th |
|---|---|---|---|---|
| 19,000 | 5,100 | 10,000 | 100 | 500 |

*Note:* CRs relate to fresh weight of mussel tissue and total water concentration. Data from [27].

*V. angasi* show behavioural responses to elevated uranium concentrations in water, with declines in duration and amplitude of valve gape and increases in frequency of valve adductions having been observed at $UO_2$ concentrations greater than 350 μg L$^{-1}$ in a synthetic Magela Creek water[42]. These valve movement responses are highly dependent on experimental conditions, with observed response endpoints varying over an order of magnitude for differing pH values and dissolved organic carbon concentrations[43].

In addition to its importance for radiological impact assessment, *V. angasi* is an important indicator species for other environmental and human health issues. These include bioaccumulation of chemical pollutants, its use in archival monitoring, and its use in laboratory and field sublethal effect studies[44,45]. As a result, this species is likely to remain a focus of efforts in monitoring and assessment of mining impact in Top End streams.

## Other Aquatic Fauna

Some earlier data for edible aquatic fauna in the ARR were published by Davy and Conway[26] and Koperski and Bywater[10]. With the exception of data for freshwater mussels, the concentrations measured were generally lower than 1 Bq $kg^{-1}$ (fresh weight) of flesh for individual radionuclides. Consequently, high-sensitivity radionuclide measurement techniques are required for studies of this nature.

Table 3 gives some summary CRs obtained from one study carried out for the Magela and Cooper Creek systems[46,47]. For most edible flesh samples, activity concentrations and CRs followed the approximate order: $^{210}Po > {}^{226}Ra > {}^{210}Pb \geq [{}^{234,238}U] \geq [{}^{230,232}Th]$.

Fig. 4 shows derived CRs for $^{226}Ra$ in the flesh of the fish barramundi (*Lates calcarifer*), plotted against the $^{226}Ra$ concentration in the water, for data obtained from four different studies: Davy and Conway (1974; ARR[26]), Davy and O'Brien (1975; Rum Jungle[48]), Akber and Hancock (1990; upper South Alligator River[49]) and Martin et al. (1995; Magela Creek system[46]). In each case the data refer to fresh weight of flesh and total water concentration. The CR values range over two orders of magnitude, illustrating the fact that, in cases where the doses are of the order of magnitude of any relevant dose limits, more robust bioaccumulation estimation methods and/or monitoring regimes will need to be used.

**TABLE 3**
**CRs (L $kg^{-1}$) for Radionuclides in Several Aquatic Fauna, Determined from Samples Collected in the ARR**

| | | $^{226}Ra$ | $^{210}Pb$ | $^{210}Po$ | U | Th |
|---|---|---|---|---|---|---|
| Fish group 1[a] | | 1,200 | 160 | 1,400 | 250 | 40 |
| Fish group 2[b] | | 190 | 35 | 180 | 15 | 22 |
| Freshwater shrimp[c] | | 270 | 39 | 1,200 | 150 | 250 |
| Turtle[d] | Flesh | 250 | 120 | 1,000 | 28 | 40 |
| | Liver | 460 | 1,100 | 38,000 | 180 | 120 |

*Note:* Ratios relate to fresh weight of tissue and filtered (<0.45 μm) water concentration. Data from [47].
[a] Fish group 1 = bony bream, sleepy cod.
[b] Fish group 2 = fork-tailed catfish, archer fish, barramundi, eel-tailed catfish, freshwater mullet, long tom, saratoga, tarpon.
[c] Freshwater shrimp = *Macrobrachium rosenbergii.*
[d] Turtle = *Elseya dentata.*

Jeffree[50] carried out laboratory-based experiments on uptake of $^{226}Ra$ by freshwater turtles, including snapping turtles (*Elseya dentata*) from Magela Creek, under varying calcium and magnesium water concentrations. He found that the capacity of *E. dentata* to accumulate $^{226}Ra$ from the aquatic medium was about two orders of magnitude less than that of tissue of *V. angasi*, in agreement with the results of the field-based studies discussed above (Tables 2 and 3). In contrast with the results of analogous experiments with the freshwater mussel *V. angasi*, for *E. dentata* increased calcium water concentrations did not significantly reduce $^{226}$Ra uptake to soft tissues (with the exception of the skin), but significantly increased the $^{226}$Ra concentration in bone and muscle. Increased magnesium water concentrations had a significant inverse effect on $^{226}$Ra uptake in bone, muscle, skin, liver, and gut, but had no significant effect for uptake to the shell.

## TERRESTRIAL FAUNA

There have been relatively few radionuclide studies on terrestrial animals in the Top End. Nonaquatic animals that have been investigated include the water buffalo (*Bubalus bubalis*), the pig (*Sus scrofa*) (both introduced species), and the magpie goose (*Anseranas semipalmata*). All three species spend much of their time in a floodplain environment.

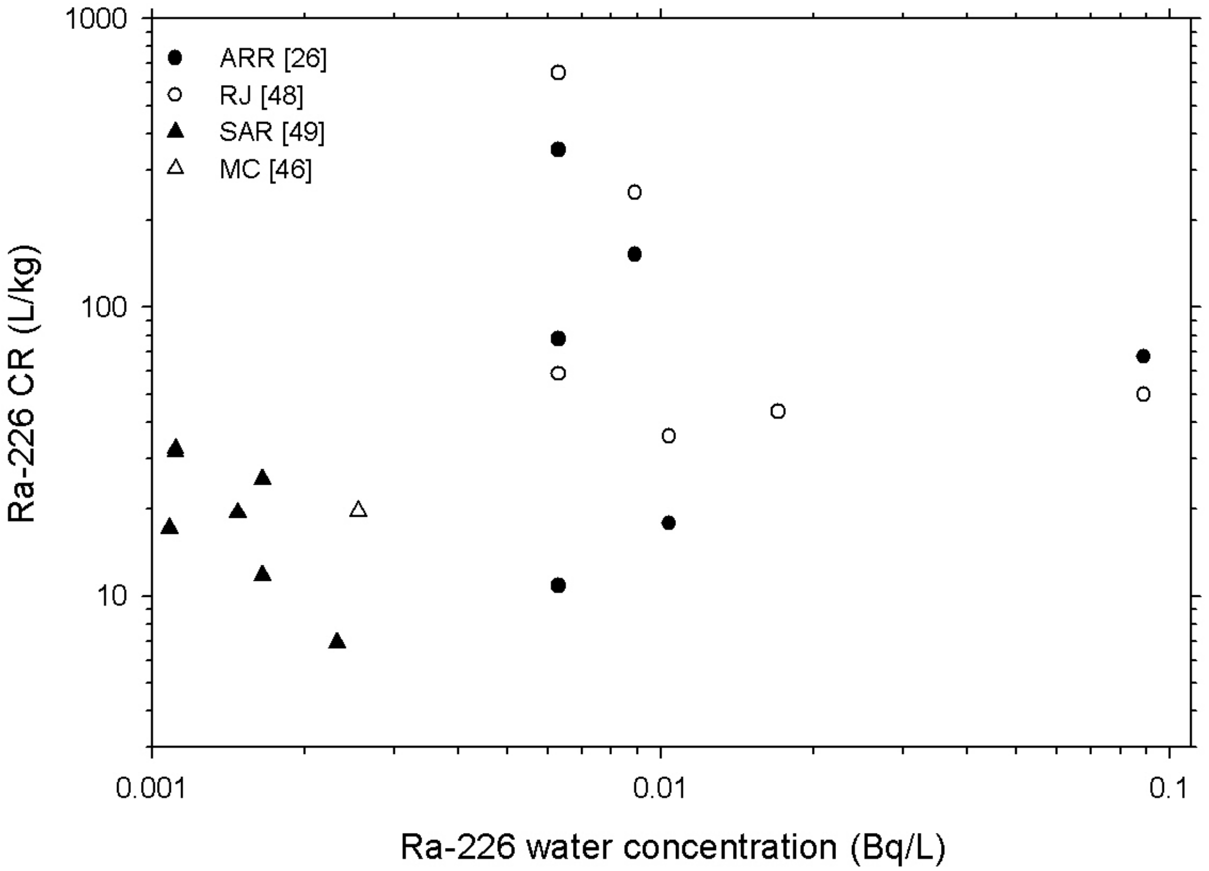

**FIGURE 4.** $^{226}$Ra CRs vs. water concentrations for samples of barramundi (*Lates calcarifer*) from four studies. ARR = Alligator Rivers Region; RJ = Rum Jungle; SAR = South Alligator River; MC = Magela Creek.

### Buffalo

The buffalo is a grazing animal with the bulk of its food coming from grass. The contribution of buffalo to Aboriginal diet has decreased markedly in recent years as a direct result of the national Brucellosis and Tuberculosis Eradication Campaign (BTEC)[51]. Tens of thousands of animals were eradicated during this program that took place from the mid 1970s and continued into the early 1990s, leaving feral buffalo numbers very low in the ARR for a number of years. Now some years after the BTEC has finished, buffalo numbers are increasing, particularly in eastern Arnhem Land[52]. Buffalo numbers are still low in Kakadu National Park[53] as a result of an active feral animal reduction program maintained by Parks Australia North. Data for uranium-series radionuclides from samples of buffalo, pig, and magpie geese that were collected in the ARR are available from several studies[10,26,46,47]. These studies showed that radionuclide activity concentrations were higher in kidney and liver than other parts of the animal, particularly for $^{210}$Po (Table 4).

**TABLE 4**
**Mean Radionuclide Activity Concentrations (Bq/kg$^{-1}$ Fresh Weight) for Buffalo, Pig, and Magpie Goose Samples Taken from the Magela Floodplain[47]**

| Material | $^{226}$Ra | $^{210}$Pb | $^{210}$Po | $^{238}$U | $^{234}$U | $^{232}$Th | $^{230}$Th |
|---|---|---|---|---|---|---|---|
| *Buffalo* | | | | | | | |
| Muscle flesh | 0.018 | 0.016 | 0.23 | 0.008 | 0.013 | 0.002 | 0.002 |
| Kidney | 0.19 | 0.14 | 19 | 0.005 | 0.011 | 0.006 | 0.007 |
| Liver | 0.043 | 0.32 | 3.2 | 0.004 | 0.002 | 0.006 | 0.005 |
| *Pig flesh* | 0.029 | 0.021 | 5.0 | 0.014 | 0.012 | 0.010 | 0.012 |
| *Magpie goose flesh* | 0.030 | 0.050 | 0.44 | 0.004 | 0.008 | 0.006 | 0.007 |

## Pigs

Early European settlers introduced pigs into Australia and it was not long before large feral populations became established. Feral pigs have succeeded across the Top End of the Northern Territory because there are good supplies of food, water, and shelter. Pigs are omnivorous and are opportunistic feeders — eating plants that include crops, fruit, and vegetables and rooting extensively for tubers, worms, and soil invertebrates — and will exploit any temporarily abundant food. They also eat animal flesh, preying on small animals as well as scavenging on carrion[54].

Feral pigs are a significant food source for local Aboriginal people[51], mainly because of their abundance and availability. Some available radionuclide concentration data are summarised in Tables 4 and 5. Both datasets show the presence of high $^{210}$Po concentrations and very high $^{210}$Po/$^{210}$Pb ratios in the edible flesh.

## Magpie Geese

Magpie geese (*Anseranas semipalmata*) are an important and popular dietary item in many Aboriginal communities in northern Australia and it has been estimated that up to 60% of Aboriginal people include magpie geese as a food source[53]. The average magpie goose can stand 70 cm tall and weigh 3 kg and during the late wet season and through the early dry season the eggs of magpie geese are consumed widely[51].

**TABLE 5**
**Radionuclide Activity Concentrations (Bq/kg$^{-1}$ Fresh Weight) for Pig and Magpie Goose Flesh Sampled During the Ranger Production Period[10]**

| | $^{226}$Ra | $^{210}$Pb | $^{210}$Po |
|---|---|---|---|
| Pig flesh | 0.02–0.1 | 0.04 | 4.4 |
| Magpie goose flesh | 0.11–1.2 | 0.41 | 3.1 |

In their study Davy and Conway[26] found values for uranium and $^{226}$Ra were higher for bones then in the flesh and liver of magpie geese. Koperski and Bywater[10] analysed flesh samples from magpie geese as part of a study of the impact of the Ranger mine, and found no significant difference between premining and mining period values. Tables 4 and 5 summarise some of the available activity concentration data[10,46]. In each case, the highest concentrations were obtained for $^{210}$Po.

## TERRESTRIAL FLORA

In the late wet and early dry seasons, vegetable foods become available and are harvested mainly by Aboriginal women. Altman[9] identified over 80 floral species used as bush foods. Although the contribution of vegetable bush foods to the Aboriginal diet has decreased in recent times due to the availability of market goods[51], these foods are still a significant potential radiological dose contributor. This is a potentially important pathway once a former minesite becomes available for public access after rehabilitation is finished.

### Fruits

Early radiological studies were conducted by Davy and Conway[26]. They reported data for uranium and $^{226}$Ra in native apples (*Eugenia* sp.) and native figs (*Ficus henneana*), and uranium in *Pandanus* palm fruit, from the ARR (Table 6).

**TABLE 6**
**Radionuclide Activity Concentrations (Bq kg$^{-1}$ Fresh Weight) for Fruit Samples[26]**

| | $^{226}$Ra | U |
|---|---|---|
| *Eugenia* sp | 0.15–15 | 0.04–0.37 |
| *Ficus henneana* | 1.1–1.8 | 0.07–0.19 |
| *Pandanus* palm fruit | | 0.02–0.30 |

Koperski and Bywater[10] published results for premining and mining period analysis for red apples (*Syzygium suborbiculare*); see Table 7. They concluded that no statistical differences were found for the specific activities of the radionuclides analysed in foods sampled during premining and mining periods.

**TABLE 7**
**Radionuclide Activity Concentrations (Bq kg$^{-1}$ Fresh Weight) for *Syzygium suborbiculare* Samples[10]**

| | $^{226}$Ra | $^{210}$Pb | $^{210}$Po | $^{230}$Th |
|---|---|---|---|---|
| Premining | 1.0–2.9 | 0.4–1.2 | | <0.08 |
| Mining | 0.3–4.0 | 0.2–0.3 | 0.7–0.8 | 0.1 |

Akber and Marten[55], in a study on radionuclide uptake by plants on the Land Application (spray irrigation) Area of Ranger Uranium Mine, reported data for two fruit samples: Cocky Apple (*Planchonia careya*) and the Grey Plum (*Persoonia falcata*); see Table 8.

**TABLE 8**
**Mean Radionuclide Activity Concentrations (Bq kg$^{-1}$ Fresh Weight) for Fruit Samples[55]**

| | $^{226}$Ra | $^{238}$U | $^{234}$U |
|---|---|---|---|
| *Planchonia careya* | 4.8 ± 0.4 | 0.05 ± 0.01 | 0.04 ± 0.01 |
| *Persoonia falcata* | 0.5 ± 0.3 | 0.24 ± 0.08 | 0.22 ± 0.12 |

*Note*: Analytical errors are one standard deviation due to counting statistics.

## Root Vegetables

Martin et al.[46] reported activity concentrations, as well as CRs relative to total soil activity, for samples of cheeky yams (*Dioscorea bulbifera*) and Anbulubi roots (*Eriosema chinense*). The reported results are shown in Table 9. The differences between the results for the two species may be partly due to the fact that the yams were peeled whereas the anbulubi were not.

**TABLE 9**
**Activity Concentrations (Above, Bq/kg$^{-1}$ Fresh Weight) and CRs (Below, Fresh Weight vs. Dry Soil Weight) for Cheeky Yams and Anbulubi Roots[46]**

| | $^{226}$Ra | $^{210}$Pb | $^{210}$Po | $^{238}$U | $^{234}$U | $^{232}$Th | $^{230}$Th |
|---|---|---|---|---|---|---|---|
| Cheeky yams | 0.26 | 0.042 | 0.21 | <0.06 | 0.06 | <0.13 | <0.3 |
| *(Dioscorea bulbifera)* | 0.014 | 0.001 | 0.007 | <0.004 | 0.004 | <0.01 | <0.015 |
| Anbulubi | 1.19 | 1.7 | 1.6 | 0.03 | 0.06 | 0.03 | 0.03 |
| *(Eriosema chinense)* | 0.055 | 0.033 | 0.031 | 0.002 | 0.003 | 0.002 | 0.001 |

## Summary

Relatively few data are available for radionuclides in terrestrial plants of the Top End, in comparison with the situation for aquatic fauna. The available data indicate that concentrations follow the following approximate order for both fruits and root vegetables: [$^{210}$Po ≈ $^{226}$Ra] ≥ $^{210}$Pb > [$^{234,238}$U ≈ $^{230,232}$Th].

## CONCLUSIONS

The research that has been undertaken into natural-series radionuclide uptake by edible flora and fauna of the Top End has mainly been conducted over the last 2 decades. As a result of the large number of possible radionuclide/food item combinations, many of the studies have concentrated on providing data suitable for calculation of CRs, although more detailed uptake studies have been undertaken for some species (freshwater mussels, turtles, and water lilies).

Despite the work that has been carried out to date, continuing study of radionuclide concentrations in Aboriginal bush foods of the region will be required for the foreseeable future, in order to provide Aboriginal people with the assurance that their food resources are being protected from mining operations. Some major areas requiring study are discussed below.

Firstly, an important factor in estimation of radiological dose is knowledge of the bushfood consumption by the relevant Aboriginal groups[9,56]. This includes knowledge of both the range and quantities of foods eaten, as well as of food preparation methods. Collection of this type of data on indigenous customary harvesting is a challenging task, particularly where quantitative data are required. This is because of the wide diversity of bush foods eaten, the fact that food collection tends to be opportunistic and often eaten at or close to the location that it is collected, and the considerable social changes occurring within the society (making the situation dynamic). As a result, it is unlikely that diet quantification will ever reach a level of reliability that is attainable for a non-Aboriginal society.

Secondly, there is relatively little available information on radionuclides in terrestrial animals and plants. Much of the available data, especially for terrestrial plants, are for concentrations in the edible portions rather than giving information on radionuclide uptake, and hence are of limited utility for predictive purposes. This information is very important for planning of uranium minesite rehabilitation, as after rehabilitation is finished the minesite may be accessed for collection of terrestrial food items. There are also few data available for bioaccumulation of radionuclides into certain other classes of Aboriginal foods, even for aquatic and semi-aquatic fauna (e.g., fats, eggs, liver, heart, bone marrow).

Finally, whereas the majority of studies to date have focussed on $^{226}$Ra and/or uranium, these are not necessarily the only radionuclides of importance from a radiological impact point of view. Table 10 shows the percentage contribution by radionuclide to committed effective dose for a 10-year-old child from a release of water from either of two of the retention ponds at the Ranger mine (these predictions are based on calculations using CRs[57] and historical data for radionuclide concentrations in the retention pond waters; pond 4 is no longer in existence but is included here as an illustrative case). Although $^{226}$Ra is the dominant contributor in the case of retention pond 2, in the case of pond 4 the predicted fraction of the dose due to $^{210}$Po is significant. In each case, the relative contribution from uranium isotopes is low due to their low CRs in most aquatic foods and relatively low dose rate conversion factors for ingestion. In the case of the Ranger mine, protection against effects on the aquatic ecosystem rather than radiological effects on humans is the most limiting factor for releases of uranium[58].

**TABLE 10**
**Predicted Percentage Contribution by Radionuclide to the Committed Effective Dose for a 10-Year-Old Child from Release of Water from**

**Either of Ranger's Retention Ponds 2 and 4[57], and from the Naturally Occurring Radionuclide Activity Concentrations**

| Radionuclide | Concentration (Bq/L) | | % Contribution | | |
|---|---|---|---|---|---|
| | RP2 | RP4 | RP2 | RP4 | Natural |
| $^{226}$Ra | 2.5 | 0.19 | 92 | 67 | 19 |
| $^{210}$Pb | 0.16 | 0.03 | 3.6 | 5.6 | 8.2 |
| $^{210}$Po | 0.02 | 0.03 | 1.5 | 25 | 48 |
| $^{234}$U | 7.9 | 0.86 | 1.1 | 1.2 | 0.02 |
| $^{238}$U | 7.4 | 0.84 | 0.9 | 1.0 | 0.02 |
| $^{227}$Ac | 0.19 | 0.014 | 0.6 | 0.4 | – |
| $^{230}$Th | 0.02 | 0.04 | 0.01 | 0.2 | 0.05 |
| $^{228}$Ra | — | — | — | — | 25 |

According to present estimates, the total committed effective dose arising from water releases from the Ranger mine are extremely low (about 0.5 μSv/year for individuals living at Mudginberri billabong[57]). By comparison, the dose received due to the naturally occurring radionuclide concentrations is estimated to be 1.6 mSv/year for an adult and 2.2 mSv/year for a 10-year-old child (this estimate is based on the Aboriginal diet assumed in [59], ICRP dose conversion factors for ingestion[60], and measured concentration data from several of the studies discussed above).

For the natural (i.e., nonmining related) dose, the greatest percentage contributor is $^{210}$Po, followed by $^{228}$Ra and $^{226}$Ra (final column of Table 10). The relatively high contribution from $^{210}$Po is due to the high dose conversion factor for ingestion for this radionuclide, and to the high concentrations observed in faunal tissue. In particular, the measured concentrations of $^{210}$Po were very high in liver, kidney, and intestine samples from buffalo and turtle, and in freshwater mussel and pig flesh. This has important consequences for the postrehabilitation scenario for the Ranger mine. In this situation, an important pathway for radiological dose will be erosion of waste rock material from the site. Unlike pond water releases, the uranium series radionuclides will be close to secular equilibrium in this material, and the relative contributions to dose by radionuclide are likely to be closer to the natural relative contributions (with the exception of $^{228}$Ra) than to those for water release. Hence $^{210}$Po is likely to be more important after rehabilitation than in the operational phase, and should be included in any future studies of radionuclide bioaccumulation in the region.

## ACKNOWLEDGEMENT

The authors wish to thank John Lowry for preparation of Figs. 1 and 3 and Kirrilly Pfitzner for preparation of Figure 2.
